# Genome-Wide Identification and Expression Analysis of the Zinc Finger Protein Gene Subfamilies under Drought Stress in *Triticum aestivum*

**DOI:** 10.3390/plants11192511

**Published:** 2022-09-26

**Authors:** Zhaoming Wu, Shenghai Shen, Yueduo Wang, Weiqi Tao, Ziqi Zhao, Xiangli Hu, Pei Yu

**Affiliations:** 1 SDU-ANU Joint Science College, Shandong University, Weihai 264209, China; 2 Marine College, Shandong University, Weihai 264209, China; 3 Research Center for Biological Adaptability in Space Environment, Institute of Space Sciences, Shandong University, Weihai 264209, China

**Keywords:** genome-wide identification, abiotic stresses, zinc finger proteins, expression pattern, drought stress, *Triticum aestivum*

## Abstract

The zinc finger protein (ZFP) family is one of plants’ most diverse family of transcription factors. These proteins with finger-like structural domains have been shown to play a critical role in plant responses to abiotic stresses such as drought. This study aimed to systematically characterize *Triticum aestivum* ZFPs (TaZFPs) and understand their roles under drought stress. A total of 9 TaC2H2, 38 TaC3HC4, 79 TaCCCH, and 143 TaPHD were identified, which were divided into 4, 7, 12, and 14 distinct subgroups based on their phylogenetic relationships, respectively. Segmental duplication dominated the evolution of four subfamilies and made important contributions to the large-scale amplification of gene families. Syntenic relationships, gene duplications, and Ka/Ks result consistently indicate a potential strong purifying selection on *TaZFP*s. Additionally, *TaZFP*s have various abiotic stress-associated *cis*-acting regulatory elements and have tissue-specific expression patterns showing different responses to drought and heat stress. Therefore, these genes may play multiple functions in plant growth and stress resistance responses. This is the first comprehensive genome-wide analysis of ZFP gene families in *T. aestivum* to elucidate the basis of their function and resistance mechanisms, providing a reference for precise manipulation of genetic engineering for drought resistance in *T. aestivum*.

## 1. Introduction

Plants may experience a variety of complex environmental stresses during their lifetime. With environmental problems such as greenhouse gases and soil pollution, abiotic stresses are becoming more common [1]. Stresses such as drought, heat, and high salinity can limit the growth and yield of crops. This phenomenon has profound implications for agriculture, forestry, and global food security issues. However, plants have evolved multiple regulatory pathways involving many transcription factor families to receive and respond to different stress signals [2,3]. Various protein families are able to regulate plant physiological processes by modifying gene expression, altering signalling, and interfering with biosynthesis to improve their adaptability [4,5,6,7].

The large and diverse zinc finger protein (ZFP) family plays a crucial role in various aspects of plant growth and development. ZFP has a highly conserved structural domain of about 30 amino acids [8,9]. As an essential motif, ZFP is involved in several physiological processes such as specific binding of proteins to DNA/RNA, protein-protein interactions, and membrane association [10]. The plant genome encodes a large number of ZFPs, the most representative being the C2H2-type, C3HC4-type, CCCH-type, and PHD-type. In addition to its role in seed germination and organ development, many studies have shown that ZFP is closely related to plants’ physiological and metabolic processes of various abiotic stress responses [11,12,13]. These protein factors are activated by stress and then alter plant tolerance through complex networks and molecular mechanisms. For instance, gene expression and crosstalk with phytohormones are directly regulated through the ABA-mediated and the mitogen-activated protein kinase (MAPK) signalling pathway [11]. For example, ZFP CCCH in *Arabidopsis* *thaliana* enhance survival by increasing the expression levels of osmoregulatory substances with enhanced expression of related stress genes [14]. The C2H2-type proteins ZFP245 and ZFP179 in *Oryza sativa* promoted the amount of free proline and soluble sugars, elevated the expression of stress-responsive genes, and enhanced their tolerance to salt and drought stresses [15]. Under cold environments, ZFP C2H2 directly regulates downstream genes to enhance cold resistance in *Sorghum bicolor* [16]. Additionally, ZFP in *Glycine soja* enhances salt tolerance by maintaining ion homeostasis and removing peroxides [17]. In summary, ZFPs play important roles in plant development and abiotic stress resistance, and in-depth studies of these proteins are necessary to understand their functions and mechanisms better.

Due to the extensive interest in their positions in the physiological regulation of resistance, some ZFP families have been identified and investigated in various plants, such as *A. thaliana*, *Solanum tuberosum*, *Pleurotus ostreatus,* and *Cucumis sativus* [18,19,20,21,22]. However, this functionally diverse family of proteins has not been studied in wheat (*Triticum aestivum*). *T. aestivum* is one of the most widely grown cereals in the world and is extensively used for industrial production [23]. It is the main ingredient in many marketed animal feeds, brewed beverages, and processed foods. The demand for *T. aestivum* as part of the global population’s diet is expected to reach over 900 million tonnes in 2050 [24]. As a result, *T. aestivum* exhibits outstanding economic and industrial value. *T. aestivum*’s breeding, cultivation, and responsiveness to the environment have been a hot research topic in plant science and agronomy. Abiotic stresses such as drought are often considered significant in limiting *T. aestivum* growth [25]. Therefore, it is necessary to study the characteristics of transcription factors that respond to abiotic stresses and thus improve the understanding of *T. aestivum* stress tolerance mechanisms. Several ZFP functions in *T. aestivum* have been reported and demonstrated for their responsiveness to abiotic stresses. However, a systematic genome-wide analysis targeting four subfamilies has not yet been performed. This is crucial for a comprehensive study of the characterization and physiological aspects of the TaZFP genes. In this study, we performed a genome-wide identification of 269 *T. aestivum* ZFPs (TaZFPs) and analyzed their phylogenetic relationships, structural features, physicochemical properties, and interactions networks. In addition, the expression pattern of the specific *TaZFP*s under drought stress was investigated using publicly available expression profiles and quantitative real-time PCR (qRT-PCR). Overall, this study provides insight into the characterization and structure of TaZFPs, lays the foundation for illustrating its biological function in response to abiotic stress, and provides theoretical support for further genetic engineering design.

## 2. Results

### 2.1. Identification and Chromosomal Distribution of the TaZFPs

Based on HMM searches of the core structural domains of the four subfamilies of TaZFPs (C2H2: PF00096, C3HC4: PF00097, CCCH: PF00642, and PHD: PF00628) and sequence alignment of ZFP gene subfamilies from *A*. *thaliana*, a total of 269 non-redundant TaZFPs were identified, which contained 9 TaC2H2, 38 TaC3HC4, 79 TaCCCH, and 143 TaPHD.

The predicted physicochemical properties of TaZFPs (Table S1) showed that, except for two sequences with a large number of unknown amino acids that could not be calculated, the length of TaZFPs ranged from 148 to 2092 aa, and the molecular weight ranged from 16.22 to 231.86 kDa. The isoelectric point of TaZFPs ranged from 4.42 to 9.85, indicating that TaZFPs contain both acidic and basic protein types. The instability index of TaZFPs varied from 35.82 to 84.75, of which only 13 protein sequences were considered stable. The aliphatic index of TaZFPs ranged from 39.08 to 102.28, and its globular protein thermal stability has diverse characteristics. According to the hydrophilicity index, 98.89% of TaZFPs are hydrophilic, with TaCCCH.76 being the strongest (−1.404) and TaPHD.108 being the weakest (−0.004). Generally, there is a diversity of physicochemical properties such as length, relative molecular mass, isoelectric point, and hydrophilicity index between the different ZFPs.

**Figure 1:**
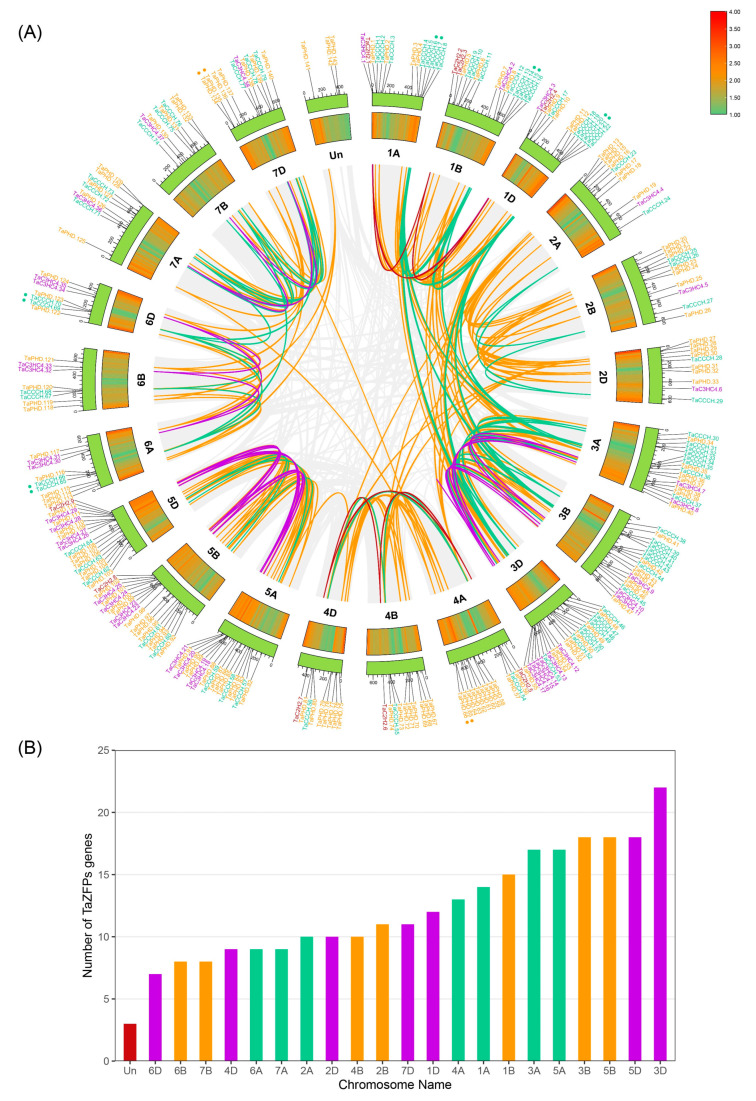
Syntenic relationships, chromosomal localization, and distribution of the *TaZFP* gene. (**A**) Red represents TaC2H2; purple represents TaC3HC4; green represents TaCCCH; orange represents TaPHD. The outer rectangles represent chromosomes and show roughly the physical location of the 269 *TaZFP* genes. The heat map of the middle rectangle indicates the gene density at the corresponding chromosome position; the grey lines in the inner background indicate col-linear blocks of *T. aestivum*; the other coloured lines indicate segmented repeat gene pairs. Tandem repeat gene pairs are located adjacent to each other and marked. (**B**) Distribution of the number of *TaZFP* genes on 21 chromosomes. Chromosomes from subgenomes A, B, and D are indicated in green, orange, and purple, respectively.

TBtools was used to create a chromosome map of the TaZFPs based on the physical location information provided by the *T. aestivum* gff3 file. *TaZFP*s were present on all 21 *T. aestivum* chromosomes and were unevenly distributed (Figure 1), whereas three TaPHD genes could not be localized to any chromosome. The specific distribution of *TaZFP*s on the chromosomes is detailed in Table S1. Meanwhile, *TaZFP* genes were equally distributed on *T. aestivum* subgenomes A, B, and D, with localization numbers of 88, 88, and 89, respectively. Chromosome 3 had the highest density of *TaZFP*s, with 57 on chromosome 3D and 17 and 18 on each of chromosome 3A and 3B, respectively. Chromosome 6D had the lowest number of *TaZFP*s, with only seven. Among the TaCCCH gene subfamilies, chromosomes 1A and 3D had the highest densities. Moreover, group 4 homologs had the lowest density and number of homologs, with almost equal numbers in the three subgenomes. TaPHD genes were distributed in higher density on chromosomes 4 and 5. On the other hand, the TaC2H2 genes could only be localized on chromosomes 5B, 5D, 1, and 4 due to the low identification number, and the TaC3HC4 gene could not be identified on chromosome 4.

### 2.2. Multiple Sequence Alignment and Phylogenetic Analysis of TaZFPs

**Figure 2:**
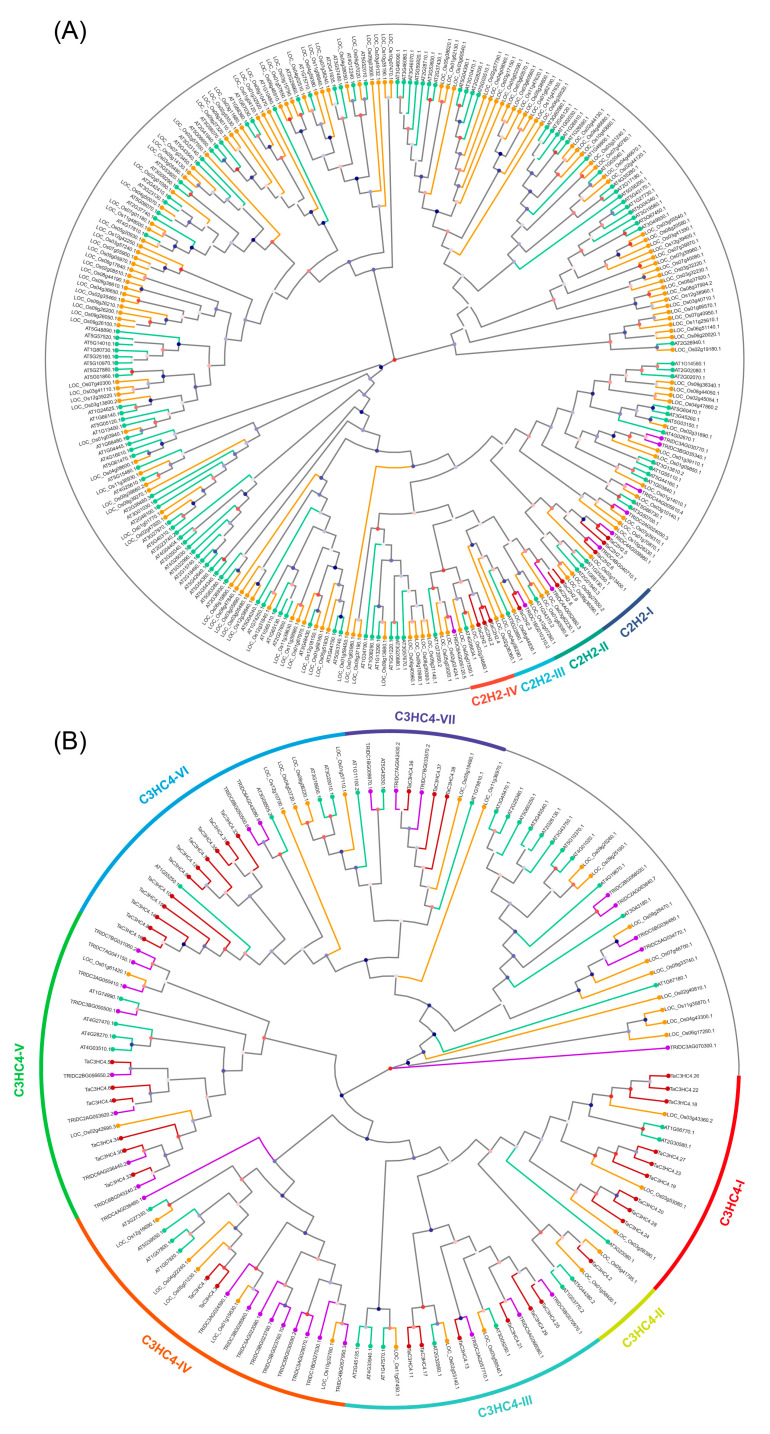

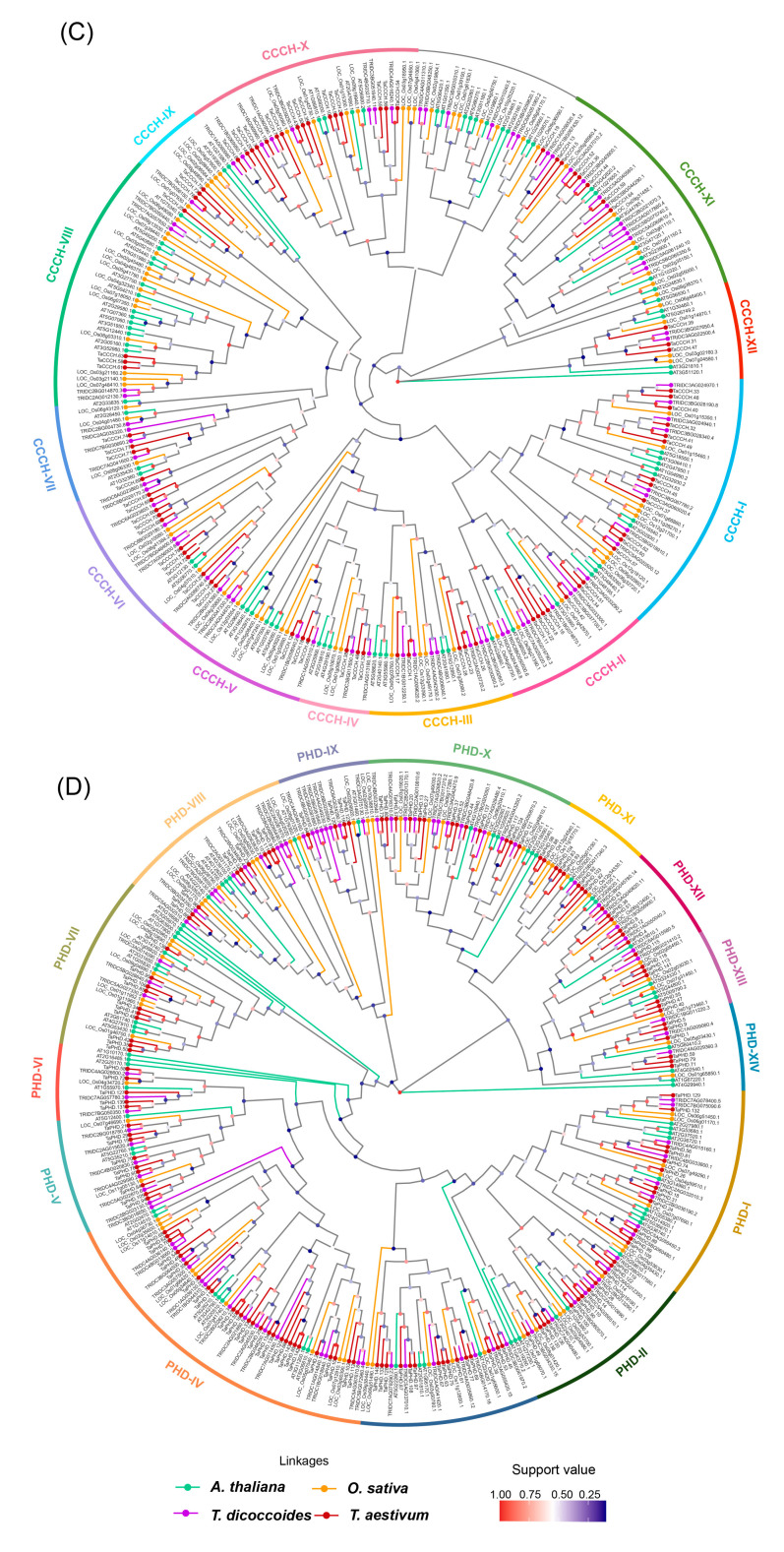
Phylogenetic trees of ZFP subfamily genes in *T. aestivum*, *T. dicoccoides*, *O. sativa*, and *A. thaliana*. (**A**) C2H2. (**B**) C3HC4. (**C**) CCCH. (**D**) PHD. Each identified subgroup is marked with Roman numerals near the branch. The different colours on the terminal branches represent ZFP genes from different species. The colours of nodes are a mapping of support values from 1000 SH tests.

To explore the evolutionary relationships of the four TaZFP subfamilies, we performed multiple sequence alignment and phylogenetic analysis of all 269 TaZFP amino acid sequences with those from *A*. *thaliana*, *Triticum dicoccoides,* and *Oryza sativa*, respectively, using the subfamilies as a taxonomic basis. After that, several unrooted phylogenetic trees were constructed using Heuristic Neighbor-Joining (HNJ) and 1000 Shimodaira-Hasegawa (SH) tests, including four subfamily gene evolutionary trees from *T. aestivum*, *A*. *thaliana*, *T.* *dicoccoides*, and *O. sativa* (Figure 2). Moreover, an evolutionary tree of gene subfamilies was constructed using all TaZFPs. The specific genes and numbers are shown in Table S2. According to the tree’s topology and the defined classification of the *A*. *thaliana* ZFP subfamilies, the phylogenetic trees of C2H2, C3HC4, CCCH, and PHD proteins from the four species were divided into several subgroups of 4, 7, 12, and 14, respectively. These results were similar to the findings of Sun et al. [26,27].

Among the identified phylogenetic subgroups, the PHD-IV subgroup contained the most significant number of TaPHD genes, accounting for 18.88% of the total number of TaPHD genes, followed by the CCCH-X subgroup containing 16 members, accounting for 20.25% of the total number of TaCCCH genes. In the C3HC4 gene phylogenetic tree, C3HC4-VI and C3HC4-I were the largest TaC3HC4 subgroups, accounting for 28.95% and 23.68%, respectively. The C2H2-III subgroup, with only one member, was the smallest taxon. In addition, there were inconsistent clusters of members of TaC2H2, TaC3HC4, and TaPHD with genes from *A*. *thaliana*, *T.* *dicoccoides,* and *O. sativa*, especially the TaC2H2 gene, that was absent in other subgroups, suggesting some changes in the C2H2 gene during the evolution of different species.

### 2.3. Gene Duplication, Synteny, and Ka/Ks Analysis of TaZFPs

Synthetic analysis is routinely used to track the evolutionary relationships of plant gene family members. Synthetic analyses were performed within the *T. aestivum* genome using MCScanX and TBtools. Table S4 shows the syntenic relationships of homologous pairs in TaZFPs. The results indicated that 243 pairs (223 *TaZFP*s) out of 269 *TaZFP*s were caused by segmental duplication, and seven pairs (14 *TaZFP*s) were caused by tandem duplication events, which are highlighted on the chromosome of Figure 2. Furthermore, fragment replication played a dominant role in the evolution of *TaZFP*s. Most of the ZFP genes in other plants were also derived from segmental duplication, such as *Gossypium hirsutum* and *Phyllostachys edulis* [28,29]. As shown in Figure 2, paralogous chromosome groups 3 and 5 contain the largest number of segmentally duplicated gene pairs. The tandem duplicated gene pairs, which mainly originate from subgenomes A and D, have the highest frequency and coverage of 91.61% in the TaPHD.

**Figure 3:**
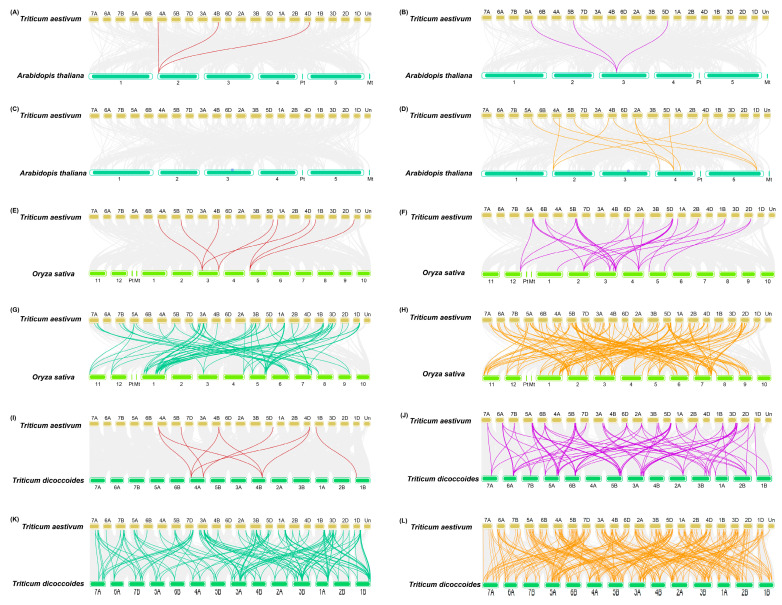
Genome-wide syntenic relationship of *TaZFP*s orthologous with three representative plants’ viz., (**A**–**D**) *A. thaliana*, (**E**–**H**) *O. Sativa,* and (**I**–**L**) *T. dicoccoides*. The grey line in the background indicates the syntenic region of *T. aestivum* and other plant genomes. The other coloured lines indicate different gene pairs, the red line indicates the co-linear TaC2H2 gene pair, the purple represents TaC3HC4, the green represents TaCCCH, and the orange represents TaPHD.

To further explore the evolutionary origins of the ZFP subfamily members between *T. aestivum* and other closely related species, we constructed a synteny map with *T.* *dicoccoides,* *O. sativa*, and *A*. *thaliana* based on orthologous gene pairs, with identified numbers 531, 239, and 20, respectively (Figure 3 and Table S5). The number of *TaZFP*s identified as homologous after de-duplication was 239, 172, and 19, respectively. In the three subgenomes of *T. aestivum*, 251 segmental duplicated gene pairs were identified (71 between subgenomes A and B, 79 between subgenomes A and D, and 75 between subgenomes B and D). This number is smaller than the number of orthologous gene pairs between *T. aestivum* and the subgenomic donor (*T. dicoccoides*).

Since the ratio of Ka/Ks is a good indicator of the selection pressure occurring at the protein level, we evaluated the values of Ks (synonymous) and Ka (nonsynonymous) as well as the ratio of Ka/Ks (Table S4). Ka/Ks < 1, Ka/Ks = 1, and Ka/Ks > 1 generally indicate negative, neutral, and positive selection, respectively [30]. A total of 240 segmental duplicated gene pairs had Ka/Ks < 1, ranging from 0 to 0.94, with mean values of Ka, Ks, and Ka/Ks of 0.06, 0.22, and 0.28, respectively. Among them, five tandem duplicated gene pairs had Ka/Ks < 1, ranging from 0.47 to 0.93, and the mean values of Ka, Ks, and Ka/Ks were 0.1, 0.16, and 0.91, respectively. The time span of the replication-derived *TaZFP*s was 0 to 128.1Mya (millions of years ago), with 76.21% of the sequences having a duplication-derived time of less than 30 Mya. This is compatible with the temporal distribution at the subfamily level. Meanwhile, the remaining part of the sequence is mainly from TaPHD and TaCCCH.

### 2.4. Gene Structure and Conserved Motifs of Four TaZFP Gene Subfamilies

We observed that intronless genes are most abundant in the TaCCCH, followed by the TaC2H2 and TaC3HC4. TaPHD.95 and TaPHD.106 contain 25 introns, the highest content among four TaZFP subfamilies. A small part of genes in the TaPHD have very large introns and very short exons, e.g., TaPHD.23 is 45,156 bp long, while the protein length is only 218 aa.

To reveal the structural variation of the TaZFPs in *T. aestivum*, The MEME program and SMART server were used to predict the conserved motifs present in TaZFPs, and a total of 12 motifs were identified (Figure S2). The results show that motif A contains Cys(2)His(2) (C2H2) residues, enabling it to form a short β hairpin and an α-helix (β/β/α structure) to bind to the DNA major groove and recognize the DNA sequence through the short α-helix [31]. The motif D with 40 to 60 residues and a cysteine spacing of C-x(2)-C-x(9-39)-C-x(1-3)-H-x(2-3)-C-x(2)-C-x(4-48)-C-x(2)-C was identified as the ring-finger conserved domain of the C3HC4 protein. The motif G has the typical C-x8 -C-x5-C-x3-H structure, which is thought to be a conserved structural domain of CCCH proteins, and motifs J and L are both typical motifs of PHD proteins. Motif K of 27 TaPHD proteins was identified as an Alfin domain that explicitly recognizes the trimethylated H3 tail on ‘Lys-4’ (H3K4me3), while other motifs were identified as conserved sequences [32]. In addition, the RRM structural domain of the bound RNA was identified in eight proteins (TaCCCH.63, TaCCCH.5, TaCCCH.58, TaCCCH.13, TaCCCH.61, TaCCCH.44, TaCCCH.25, TaCCCH.52). A large number of domains related to histone binding, DNA binding, and chromatin activity regulation were also identified in TaPHD, such as BAH, BRCT, ING, HOX, SET, SAP, etc. Notably, TaC2H2 and TaCCCH are commonly present with multiple characteristically conserved domains with a repeat count of 2-7 and a few TaPHDs (TaPHD.24, TaPHD.35, TaPHD.50, TaPHD.70, TaPHD.74, TaPHD.126, TaPHD.130, TaPHD.138, etc.) also have the phenomenon, where the number of repetitions is three. This duplication and extension of the finger-like structural domain contribute to DNA-zinc finger protein interactions [33,34]. Overall, the *TaZFP*s have high structural intron diversity, and the corresponding protein identifies the zinc finger motif.

### 2.5. Stress-Related Cis-Acting Regulatory Elements (CAREs) of the TaZFP Gene Promoter

**Figure 4:**
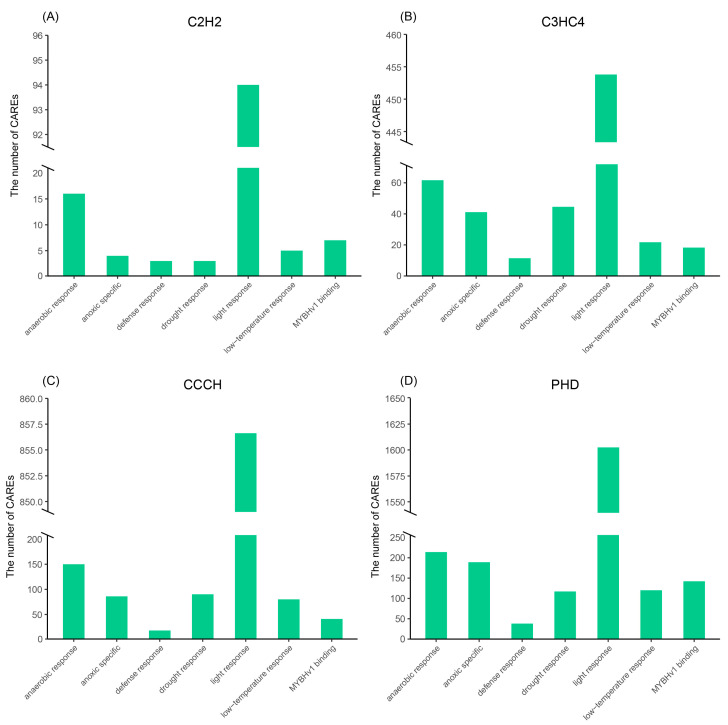
The most common *cis*-acting regulatory elements of the different *TaZFP* subfamilies. (**A**) TaC2H2 (**B**) TaC3H4 (**C**) TaCCCH (**D**)TaPHD.

The 2kb upstream promoter region of four TaZFPs subfamilies members was extracted and analyzed using PlantCARE online software to investigate their evolutionary and functional differences. A total of 40 abiotic stress response-related Cis-acting regulatory elements (CAREs) were identified in the *TaZFP*s (Table S6), with TaC2H2, TaC3HC4, TaCCCH, and TaPHD predicting 26, 30, 35, and 37 motifs, respectively. CAREs involved in the light response are most abundant and prevalent in the *TaZFP* gene subfamily (Figure 2). These genes are upstream of multiple CAREs (e.g., G-box, P-box) that bind transcription factors (e.g., MRE) and phytohormones. This suggests that *TaZFP*s may be cross-regulated with other proteins and play a crucial role in light responses that are co-regulated by phytohormones and multiple transcription factors. In addition, many CAREs are involved in abiotic stress or environmental stress response processes such as low temperature, hypoxia-specific induction, and anaerobic induction, such as ARE, DRE, GC-motif, and LTR [35,36,37,38]. The numbers of these are shown in Figure 4. These environmental stress-related CAREs were found in the promoter regions of the ZFP genes of the C2H2 type in *S*. *tuberosum*, the CCCH type in *Solanum lycopersicum*, the C3HC4, and the PHD type in *Capsicum annuum* [13,39,40,41]. The distribution of CAREs on genes is shown in Figure S3.

### 2.6. Gene Ontology (GO) Enrichment and Protein-Protein Interaction Network of TaZFPs

**Figure 5:**
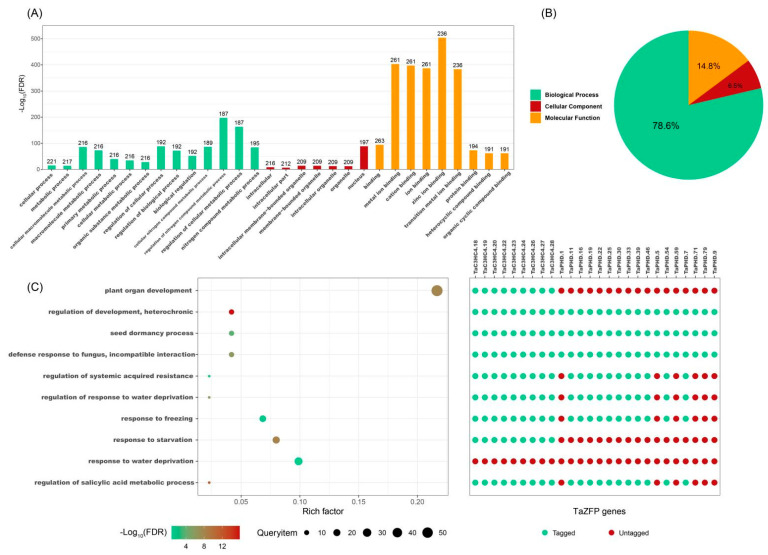
Gene ontology (GO) enrichment analysis of 269 *TaZFP*s under drought stress. (**A**) Top thirty most annotated GO items. (**B**) The proportion of different GO annotations. The cut-off value is FDR ≤ 0.05 for 501 GO entries. (**C**) Representative TaZFPs are assigned to categories related to development, stress, and hormone response and regulation. Green circles indicate that the gene does not belong to this category; red circles indicate that the gene belongs to the relevant category; the colour gradient indicates the size of the -Log10 (FDR) value; the size of the circle indicates the number of TaZFPs; the ‘Rich factor’ refers to the number of TaZFPs as a proportion of the total number of genes.

All *TaZFP*s were successfully annotated and assigned GO terms (Table S7). The enrichment results of the agriGO database showed that the annotation of the biological process category was the central part. The GO terms in the biological process category relate to processes such as growth and development, stress response, and hormone regulation, including plant organ development (GO:0099402), regulation of development, heterochronic (GO:0040034), response to water deprivation (GO:0009414), regulation of response to water deprivation (GO:2000070), regulation to freezing (GO:0050826), and regulation of salicylic acid metabolic process (GO:0010337). In the molecular function category, the majority of the *TaZFP* subfamily was enriched in zinc ion binding (GO:0008270), followed by a majority enriched in nucleic acid binding (GO:0003676) (Figure 5). The cellular component category showed enrichment mainly in the intracellular membrane-bounded organelle (GO:0043231) and nucleus (GO:0005634). Similar GO annotation content was observed for the ZFP genes of *C*. *sativus*, *Brassica napus,* and *C. annuum* [22,41,42].

We selected 10 representative biological processes shown in Figure 5 below, including growth and development, stress response, and hormone regulation. The 26 proteins with response to water deprivation annotation were searched in the other nine biological processes to see whether they simultaneously functioned in more than one biological process. The results showed that 17 of the 26 proteins involved in response to water deprivation possessed both regulation of response to water deprivation, i.e., both response and positive regulation of response to water deprivation [43]. At the same time, these 17 proteins are also involved in plant organ development. In regulating systemic acquired resistance, regulation of water deprivation, response to freezing, and regulation of the salicylic acid metabolic process, there are six identical proteins, TaPHD.1, TaPHD.5, TaPHD.9, TaPHD.59, TaPHD.71, and TaPHD.79. It is tentatively assumed that these six proteins will likely play essential roles in growth and development, stress response, and hormone regulation. However, it should be noted that the 26 selected proteins were not associated with the regulation of development, heterochronic, and GO records.

**Figure 6:**
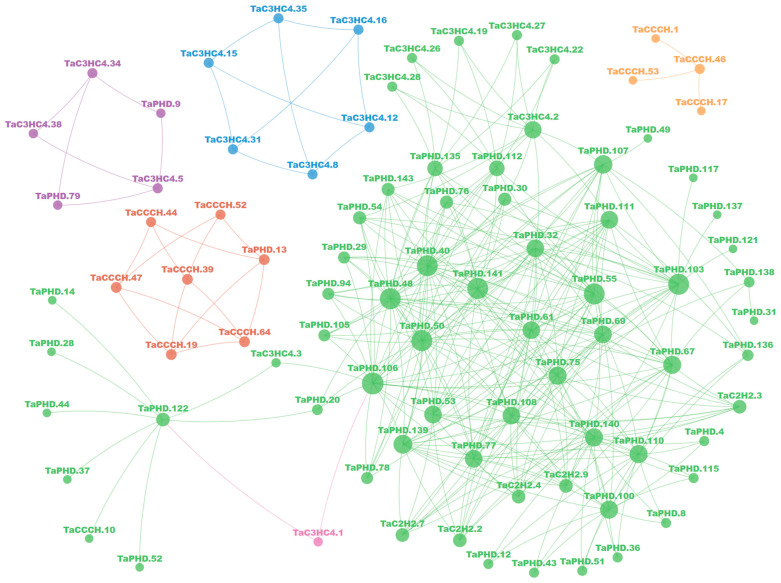
Putative interaction network of TaZFPs in *T. aestivum*. A total of 366 interactions are shown between 88 TaZFPs. The size of the circle is a mapping of the number of interacting objects. The colour of the circle represents the clustering category to which it belongs.

Protein interaction analysis and hierarchical clustering results showed that eight TaZFPs interact in six classes and five relatively independent networks, with members of all four ZFP subfamilies involved (Figure 6). TaPHD members, which occupy the central part of the most complex network, still play an important role, with a total of 23 proteins covering four ZFP subfamilies interacting and acting on more than 10 objects. Among them, there are several proteins with identical targets, e.g., TaPHD.4, TaPHD.8, TaPHD.12, TaPHD.36, TaPHD.43, TaPHD.51, and TaPHD.115 all interact with their nearby TaPHD.100, TaPHD.110, and TaPHD.140.

### 2.7. Expression Pattern Analysis of TaZFPs under Abiotic Stresses

**Figure 7:**
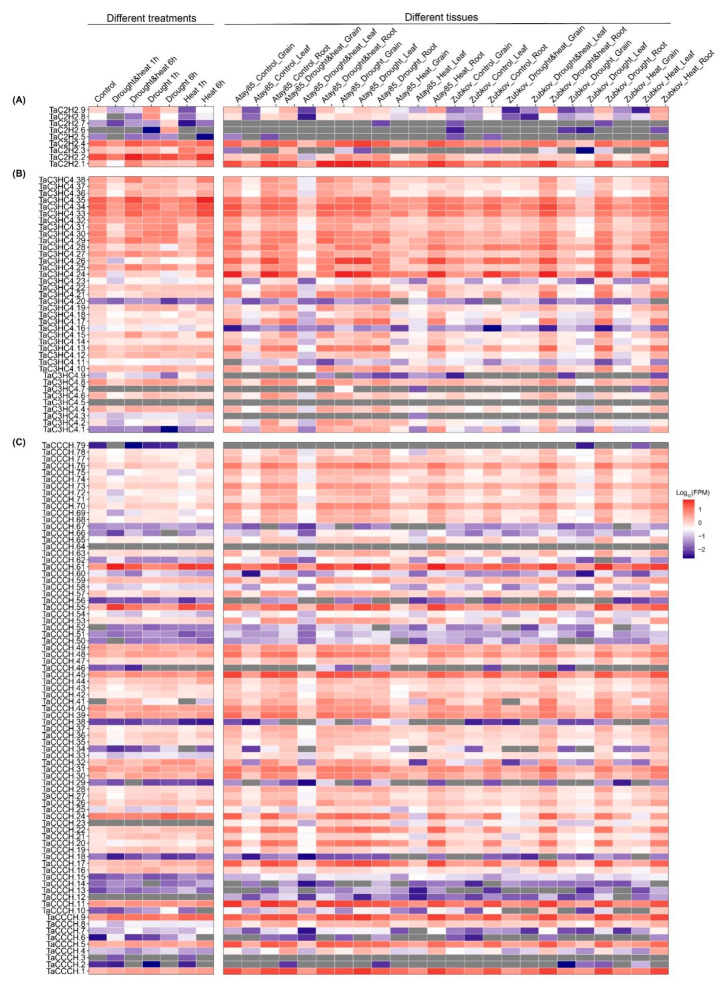

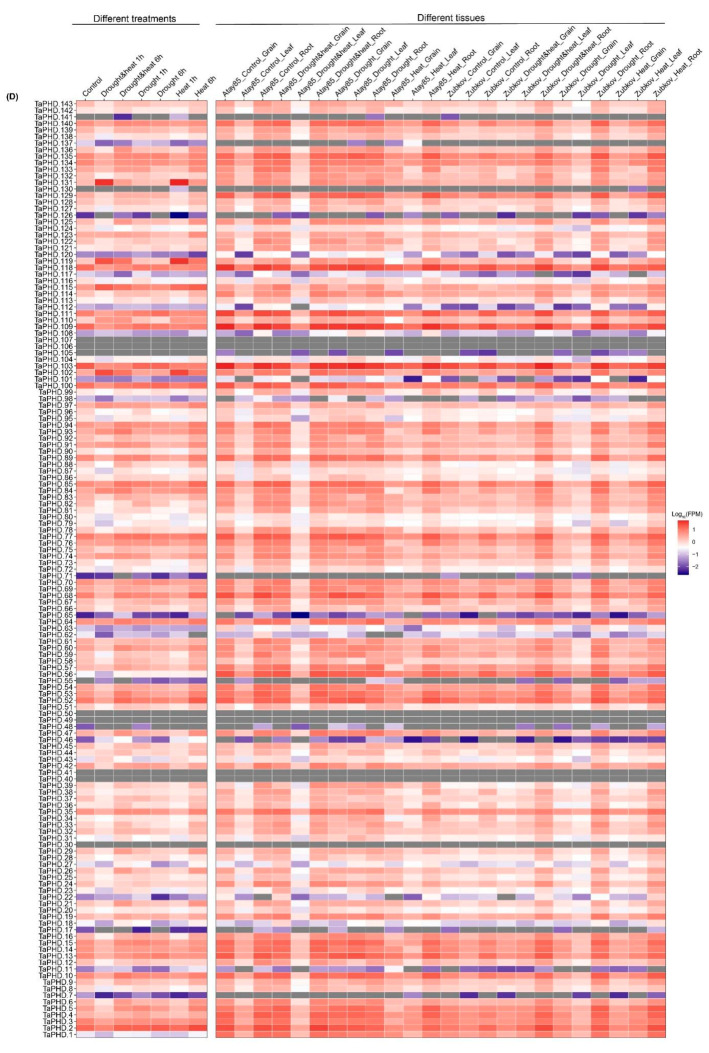
TaZFP gene expression levels heatmap at different treatment times and tissues under drought stress. TPM values were scaled by the logarithm of the base 10 to form the heatmap. (**A**)TaC2H2 (**B**) TaC3HC4 (**C**) TaCCCH (**D**) TaPHD.

To investigate the tissue-specific expression patterns and functions of *TaZFP*s in *T. aestivum* under abiotic stress, we performed expression profiling in different tissues under drought stress (DS) and heat stress (HS) treatments (Figure 7 and Table S8) [44]. The results showed that most *TaZFP*s were highly expressed in seedling leaves while exhibiting tissue-specific expression patterns that were detectable in leaves, roots, and grains, except for 17 genes that were not expressed within either data set. Genes with little or no expression were concentrated in the TaC2H2-I, TaCCCH-X, TaPHD-IV, and TaPHD-XI subgroups. Meanwhile, some members of TaC3HC4-V, TaCCCH-I, TaCCCH-III, TaCCCH-XI, and TaPHD-IV were consistently expressed at high levels in all three tissues. Among them, the TaPHD-IV subpopulation contained very low or no expression and higher expression levels of the members. In *T. aestivum* seedling leaves, the expression of 149 genes increased after DS-1h, and 53 of them (e.g., eight genes from TaCCCH-I, six from TaCCCH-VI, six from TaCCCH-X, etc.) continued to increase at DS-6h. Whereas 52 genes were up-regulated under both HS-1h (eight PHD-III, seven CCCH-X, etc.) and DS and HS-1h (eight PHD-III, six CCCH-X, etc.), and there was an overlap of 41 genes (six PHD-III, six CCCH-VI, six CCCH-X and all five members from CCCH-III, etc.). After 6 h, the number of genes up-regulated under HS and DS, and HS treatment increased, and all 139 were up-regulated (all 13 members from PHD-X, 11 PHD-III, 10 PHD-VI, etc.). Meanwhile, a significant amount of ZFP genes were down-regulated under DS treatment relative to the susceptible variety Atay85. Only 27 of these genes were up-regulated in expression, mainly involving PHD and CCCH genes, and did not contain members of the C2H2 subfamily. The resistant variety Zubkov had 191 genes with up-regulated expression under the leaves (e.g., TaCCCH-I, TaPHD-IV, TaC3HC4-I, TaC3HC4-VI, and most subgroup members). This marked difference in expression was also present in root tissue. Varietal expression pattern differences in the grain remained. In contrast, the number of genes up-regulated in response to expression was much higher in the grain of Atay85 than in that of Zubkov. The differential expression patterns of *TaZFP*s under HS and DS, and HS treatments were also similar in root and grain tissues of both species. However, most genes were up-regulated in leaf tissues under DS and HS treatment, and the level and number of genes up-regulated were greater in Atay85 than in Zubkov.

**Figure 8:**
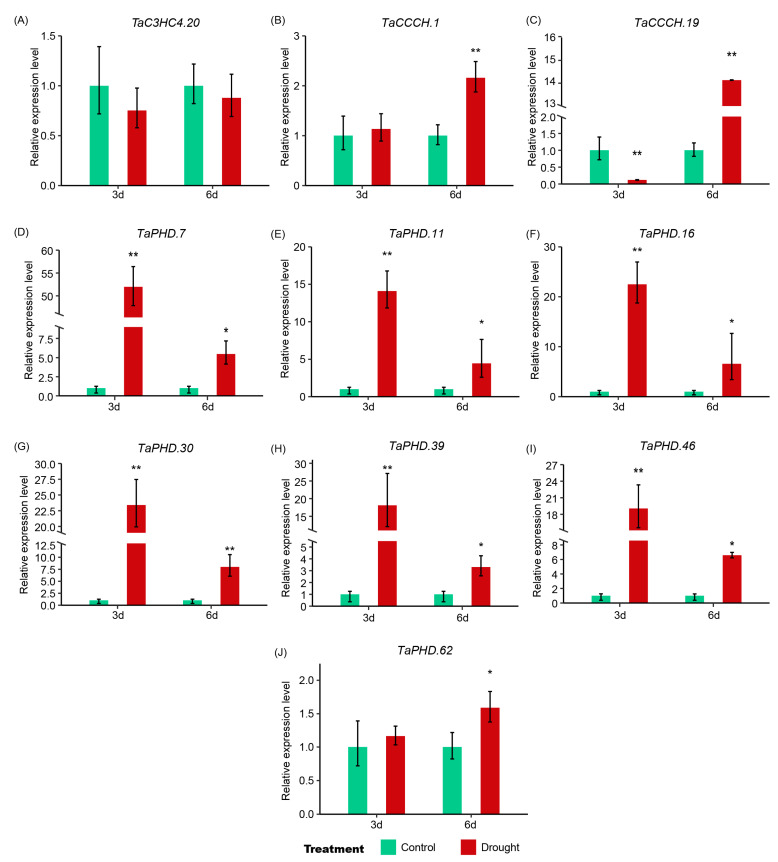
The relative expression levels of *TaZFP* genes after 3-day (3d) and 6-day (6d) drought treatments were examined by qRT-PCR. The *T. aestivum* actin gene was used as an internal reference control. Relative expression levels are the mean ± SE of the three samples, with significant differences marked as (*) *p* < 0.05 and (**) *p* < 0.01 under the *t*-test. (**A**) TaC3HC4 (**B**) TaCCCH.1 (**C**) TaCCCH (**D**) TaPHD.7 (**E**) TaPHD.11 (**F**) TaPHD.16 (**G**) TaPHD.30 (**H**) Ta PHD.39 (**I**) Ta PHD.46 (**J**) Ta PHD.62.

To confirm expression patterns of *TaZFP*s from transcriptome data, 10 *TaZFP*s were selected based on GO enrichment and expression profiling results. Their expression levels in seedling leaves under drought treatment were analyzed using qRT-PCR experiments (Figure 8). Except for *TaC3HC4.20*, the relative expression levels of nine genes were increased in DS-3d and DS-6d treatment. Among them, six genes from the PHD-VI subgroup (Figure 8D–G,I) showed similar differential expression patterns. Their relative expression levels increased by at least 10 times under DS-3d treatment and about 5 times after DS-6d treatment. The expression levels of *TaCCCH.1* and *TaPHD.62* were the same as those of reference genes under DS-3 d but increased at DS-6 d. Based on the results of qRT-PCR, the expression profiles of the 10 *TaZFP*s were generally consistent with previously published data.

## 3. Discussion

ZFP transcription factors are widespread in the plant kingdom and play an instrumental role in plant growth, development, and response to environmental stress. ZFPs have been identified in many plant species [5,45,46]. However, no systematic studies have been reported on the four TaZFP subfamilies of *T. aestivum*. As a widely grown cereal crop, *T. aestivum* has significant economic and agricultural values. Uncovering the characterization of the *TaZFP* gene family under abiotic stress is essential for further understanding and improving the resistance of *T. aestivum*. In this study, 9 TaC2H2, 38 TaC3HC4, 79 TaCCCH, and 143 TaPHD were identified. Subsequently, their physicochemical properties, phylogenetic relationships, gene duplications, gene structure, conserved structural domains of peptide sequences, stress-related CAREs, GO annotation, protein-protein interactions, and expression profiles under DS and HS were analyzed.

Physicochemical properties are these functional proteins’ most fundamental and crucial characteristics [26]. We have analyzed the physicochemical properties of TaZFPs and predicted the protein sequence’s length, molecular size, and isoelectric point. The projections are consistent with the results for *S. lycopersicum* and *P*. *edulis* [39,47]. The higher hydrophilicity may be related to these proteins’ high number of repeats and the need for a finger-like structural domain for binding to nucleic acids. Four subfamilies have high diversity in length, isoelectric point, and instability, suggesting they may have a diverse functional subgroup classification. These polymorphisms within four gene subfamilies may be a prerequisite for the diversity of physiological functions in which TaZFPs are involved, i.e., the existence of differential responsive regulation by different members of the same subfamily [11,12].

The chromosomal localization results showed that *TaZFP*s were evenly distributed across the three subgenomes of *T. aestivum* but at varying densities on the chromosomes. On the subgenomic levels, this may lead to genes with redundant functions, suggesting that some *TaZFP*s may have undergone gene loss events during evolution, subjecting to low purifying selection. Meanwhile, this uneven distribution of ZFP is also typical in other plants such as maize and *S. lycopersicum* [39,48]. Some studies attribute this differential localization to gene duplication patterns. As a result, genes from the same family are distributed in different chromosomes to achieve full function [49].

Based on phylogenetic analysis, TaC2H2, TaC3HC4, TaCCCH, and TaPHD proteins were classified into 4, 7, 12, and 14 subgroups, respectively. The distribution of motifs of members of the same subfamily tends to be more highly conserved. The differential grouping of motif composition combined with the results of phylogenetic analysis supports the reliability of group classification and suggests that *TaZFP*s in different groups may be functionally divergent. The results of the *TaZFP* evolutionary branching of *TaZFP* are consistent with four *TaZFP* subfamilies’ phylogenetic clustering, further supporting the evolutionary relationship between each TaZFP member. It has been reported that the functions of homologous genes can be inferred from the phenotypes of highly homologous genes from *A.* *thaliana* [50,51]. The 31 groups in our study (37 groups in total) are supported by previous high introductory studies (Table S3). For example, DRIP1 and DRIP2, which are homologous to nine members of the C3HC4-I subgroup, are involved in regulating stress-related transcriptional changes and drought resistance. In *A*. *thaliana*, atU2AF35b and atU2AF35b, which are orthologous to the CCCH-XI subgroup, are important splicing factors localized in the nucleus and cytoplasm as U2 auxiliary factor small subunits and have a vital role in recognition of the 3’-splice site [52]. Altered expression levels can lead to pleiotropic traits such as late flowering, abnormal leaf morphology, and flower and angular fruit shape [53]. On the other hand, whether C3HC4-I and CCCH-XI subgroups are drought-resistant needs to be further verified.

It is worth mentioning that all TaC2H2s are located in the same large branch in the C2H2 protein evolutionary tree and are missing in other branches (Figure 2). Genes clustered in branches of the phylogenetic tree are generally considered to be conserved in their evolutionary status [26]. Considering that this conserved feature is also found in plants such as *Camellia sinensis*, *O. sativa*, etc., it can be assumed that TaC2H2 has evolutionary conservatism [54,55]. Furthermore, consistent with the evolutionary closeness of the four species, ZFP genes from *T.* *dicoccoides* were the most clustered with *TaZFP*s, followed by *O. sativa*. Regarding the number of *ZFP*s, it was found that the number of genes in *A*. *thaliana* and *O. sativa* was 26 and 35, respectively, more than that in *T. aestivum*, while the number of genes in *T. dicoccoides* was 64. Among them, the difference in the number of C3HC4 and CCCH genes between species was slight, while the number of C2H2 genes in the other three species was much more than that of *T. aestivum*, and the number of PHD genes was significantly less than that of *T. aestivum*. Despite the considerable variation in genome size and the number of genes encoded between the four species, the number of gene subfamilies encoding CCCH is similar. This may be because some of the CCCH genes became highly functionally differentiated during expansion and evolution earlier than the differentiation time in monocots and dicots [56]. In comparison, PHD members showed substantial expansion and functional divergence relatively late in most subgroups. The four *TaZFP* gene subfamilies in *T. aestivum* have been subjected to differential selection for intensity during evolution, and further gene duplication analysis is required.

Gene duplication events are an important source of gene novelty and a significant influence on the expansion of gene families that aggregate into gene families that facilitate the adaptation of plants to a wide range of current environmental challenges. There are four types of duplication: segmental duplication, tandem duplication, whole genome duplication, and trans-poson-induced duplication [57,58]. Gene duplication analysis showed that the fragment repeats that had dominated the evolution of the four TaZFP subfamilies, in particular, have contributed significantly to the massive expansion of the TaPHD family in *T. aestivum*.

Moreover, the syntenic analysis revealed that *T. aestivum* has varying degrees of orthologous relationships with members of the ZFP subfamily of three other closely related species. Among them, the number of orthologous gene pairs between *T. aestivum* and *T.* *dicoccoides* even exceeded the number of paralogous gene pairs between subgenomes of *T. aestivum*. Given the high degree of evolutionary homology between the two species and the greater number of duplicated genes compared to those within the subgenome, it can be inferred that this phenomenon is associated with polyploidy and gene loss, or chromosomal recombination, during evolution [59,60]. In addition, the orthologous genes of 14 *TaZFP*s in *T. aestivum* were observed in the other three species, indicating that these genes are relatively conserved during evolution. Nevertheless, in addition to 14 evolutionarily highly conserved orthologous genes identified in all four species, some of the identified TaZFPs are only synthetically related to genes in one species. For example, 24 *TaZFP*s were covalently related to *T. dicoccoides* (C2H2:1, C3HC4:3, CCCH:9, PHD:11). This implies that *TaZFP*s, especially PHD and CCCH, may have been lost and retained in the remaining two plants in *T. aestivum* and *T. dicoccoides*. Furthermore, this phenomenon could further explain the high homology of ZFP genes between them. Additionally, the number of paralogous gene pairs corresponding to the same gene between genomes in *T. aestivum* was generally 1-2 times. In comparison, it was maintained mainly above 3 times in the other three species, suggesting that four TaZFP subfamilies may have undergone a stronger selection pressure during its evolution in *T. aestivum* [45].

Paralogous and orthologous synthesis analyses suggest that the evolution of TaZFPs is dominated by segmental duplication events and may be subject to strong selection. Segmental duplex gene pairs account for a significant proportion of the evolution of the four TaZFP gene subfamilies. The results for Ka/Ks further validate that most duplicated genes, especially segmentally duplicated gene pairs, underwent strong purifying selection to reduce deleterious mutations, thus maintaining this gene subfamily’s size and possible expression. This is consistent with the results of other plants, such as *Brassica rapa* and *Panax ginseng* [33,61].

The structure of a gene determines its coding potential and can also suggest the ancestry of the gene. This is because structurally similar genes may have evolved from a common ancestor [62,63]. Introns are essential components of genes and play an important role in gene expression regulation and stabilization through intron-mediated enhancers, selective splicing, and increased efficiency of natural selection [64]. What can be found is that most *TaZFP*s have introns and a high diversity of features, such as the number, length, and distribution of introns (Figure S1), which may be caused by intron deletion and insertion events. Deshmukh et al. showed in rice that the mRNA produced by transcription of intron-rich genes might have higher stability [65]. The expression levels of these genes with long introns may be positively affected.

*Cis*-acting regulatory elements (CAREs) are short DNA sequences found on promoters. They regulate gene expression and are essential to the gene regulatory network. On the other hand, these non-coding DNA fragments also provide an idea about physiological processes, suggesting specific genes that may be involved [66,67]. For instance, PbrMYB21 can interact with the MYB-recognizing *cis*-element in the promoter region of PbrADC to regulate polyamine synthesis by modulating ADC expression levels, thereby altering the drought tolerance of *Pyrus betulaefolia* [68]. The observation of these CAREs by four *TaZFP* gene subfamilies suggests that TaZFP plays a vital role, mainly in light and abiotic stress responses. The importance of light in plant growth and development has been demonstrated. It is proposed to influence light-regulated trans-acting factors through the transduction of light signals and its binding to cis-acting elements in gene promoters to facilitate transcription in response to light. Directly or indirectly, this abiotic factor regulates the development and differentiation of plant cells [69,70]. In this study, a total of 32 species of 2927 light response-related CAREs identified from the upstream 2 kb fragment of the *TaZFP gene* indicate the critical involvement of *TaZFP*s in plant growth and development. Meanwhile, a total of 249 MBS were identified for 143 *TaZFP*s, revealing that the transcription factor MYB is also involved in regulating drought inducibility [71]. Overall, many *cis*-acting elements associated with environmental stress and plant hormone responses were identified, suggesting that the four *TaZFP* gene subfamilies may be involved in multiple signalling pathways [72,73].

GO analysis provides the possible basic functions of genes in cells, i.e., biological processes, molecular functions, and cellular components. It has been widely used to determine the various functions of plant and animal genes [74]. Overall, these GO terms highlight four subfamilies of TaZFPs that function primarily in the nucleus and organelles within the cell membrane. They bind to nucleic acids through finger-like structures formed by chelation with Zn^2+^ and are involved in regulating several processes such as growth and development, abiotic stress responses, and hormonal regulation.

Protein-protein interaction prediction can reveal putative relationships among proteins. Interacting proteins may play important roles in plant growth, development, and response to various stresses through integrative regulation [75,76]. The interaction network shows several proteins with a high number of interactions. Among them, the Inhibitor of Growth (ING) transcription factors (TaPHD.100 and TaPHD.110) can bind unmodified to the lysine 4-trimethylated histone H3 (H3K4me3) [77]. Furthermore, TaPHD.140 with the PHD, JmjC, and PLU-1 structural domains may play a role in the histone demethylation machinery. They are hypothesized to play a key role in regulating functionally diverse protein networks [78].

Tissue-specific expression differences are one of the most critical indicators of functional differentiation among genes and facilitate the regulation of various physiological processes by eliminating their redundancy [79]. The expression profiles suggest that most TaZFPs are involved in multiple plant growth and development processes and have tissue-specific or preferential expression patterns. In particular, members from the TaC3HC4-V, TaC3HC4-VI, TaCCCH-I, TaCCCH-III, TaCCCH-VI, TaCCCH-X, TaCCCH-XI, TaPHD-III, TaPHD-IV, TaPHD-X, and TaPHD-VI subgroups. The level of TaZFPs expression was more positively regulated in leaf and root tissues under both abiotic stresses, DS and HS, than in grain tissues.

Drought is one of the important environmental stressors affecting *T. aestivum* growth and development, often leading to reduced *T. aestivum* yield [80]. Previous studies have shown that the ZFP*s* respond to various abiotic stresses. For example, double mutants of AT1G06770.1 (DRIP1) and AT2G30580.1 (DRIP2) are more tolerant to drought stress compared to wild-type *A. thaliana* [81]. *TaC3HC4.20* is orthologous to *Arabidopsis* DRIP1 and DRIP2 and is down-regulated under drought-6d treatment, suggesting that they may have similar functions under drought stress. They are capable of mediating DREB2A ubiquitination and targeting 26S proteasomal protein hydrolysis. Under dehydration treatment, they act as E3 ubiquitin ligases with reduced expression levels, delaying DREB2A-regulated drought-responsive gene expression and negatively regulating drought-responsive gene expression for seed germination and seedling growth while improving drought tolerance in transgenic *A. thaliana*. Moreover, *TaC3HC4.20* was found to have a *cis*-element MBS for drought-induced stress response, which supports our hypothesis [82]. Notably, The expression level of *TaCCCH.1* showed a 14-fold increase under 6-day drought treatment. *TaCCCH.1* is a member of the CCCH-XI subgroup and is homologous to atU2AF35b and atU2AF35b, which are key factors in recognizing the 3’ shear site of the mRNA precursor [53]. Therefore, this phenomenon suggests that it plays an important role in maintaining physiological processes such as plant growth and development under drought stress. Overall, these results validate the involvement of TaZFPs in plant responses to drought stress.

## 4. Materials and Methods

### 4.1. Identification of TaTFPs in Triticum aestivum

The structural domain and sequence identification were used to identify the TaZFP gene subfamilies in *Triticum aestivum*. First, the protein family ID (C2H2: PF00096, C3HC4: PF00097, CCCH: PF00642, PHD: PF00628) corresponding to the structural domains of each of the four ZFP gene subfamilies was retrieved from Pfam (http://pfam.xfam.org/, accessed on 3 May 2022), and the Hidden Markov Model (HMM) files for all protein subfamilies were downloaded. All protein sequences of *T. aestivum* were obtained from the ensemblPlants database (https://plants.ensembl.org/index.html, accessed on 5 May 2022) and used to perform HMM searches (E-value < 0.01) on the software TBtools against the local protein database [26,83]. The search results were initially screened by taking the same part of the sequence score and the domain score. For determination, sequences of the four AtZFP gene subfamilies of *A. thaliana* were obtained directly from the Phytozome database (https://phytozome-next.jgi.doe.gov/, accessed on 6 May 2022) [84]. The sequences were used as reference sequences for Blastp (E-value < 10^−10^) to confirm the homologous sequence in *T. aestivum*, which are potential *TaZFP* gene subfamily candidates.

In summary, based on the results of the above two approaches, the NCBI CDD (https://www.ncbi.nlm.nih.gov/Structure/cdd/wrpsb.cgi, accessed on 8 May 2022) and the HMMscan (https://www.ebi.ac.uk/Tools/hmmer/search/hmmscan, accessed on 8 May 2022) were used to confirm the presence of the core domain of TaZFPs. Finally, the protein sequences were examined by manual screening, mainly by running Blastp on NCBI (https://www.ncbi.nlm.nih.gov, accessed on 13 May 2022) and Uniport (https://www.uniprot.org/, accessed on 13 May 2022). The above processes were performed with redundancy removal operations. The gene subfamily localization of TaZFP on chromosomes was obtained from the EnsemblPlants database. The names of Ta and corresponding ZFP gene subfamilies were used as naming prefixes. Then, the numbering was added in order of the position of the gene subfamilies on the chromosomes from the long arm to the short arm, respectively. The ExPASy-ProParam online tool (https://web.expasy.org/protparam/, accessed on 28 June 2022) is used to predict the physicochemical properties of TaZFPs.

### 4.2. Multiple Sequence Alignment and Phylogenetic Analysis

Multiple sequence alignment of genes from four species, *T. aestivum*, *A. thaliana*, *O. sativa,* and *T. dicoccoides*, was performed separately according to the four ZFP subfamily categories using ClustalW on Mega-X software. All analyses use the software’s default parameters. Then, sequence alignment was trimmed by TBtools v1.098 (Chen, C.C., South China Agricultural University (SCAU), Guangdong, China) as input files for the FastTree program v2.1.11 to construct Heuristic Neighbor-Joining trees. After that, the Jones-Taylor-Thorton (JTT) model of amino acid evolution and 1000 times SH tests were performed in the process [85,86].

### 4.3. Determination of Chromosome Distribution, Synteny, and Ka/Ks of 4 TaZFP Gene Subfamilies

Chromosomal location information and the tandem and segmental duplication of the *TaZFP*s were analyzed using TBtools, the multicollinearity scanning tool MCScanX and BLASTP [87]. Data were obtained from the genome of *T. aestivum* in EnsemblPlants and gff3 annotation files. Afterward, the chromosomal distribution, intra-, and inter-specific (with other 3 species) gene synteny of *TaZFP*s were visualized. Duplicate events with ≥80% sequence similarity were identified by the bidirectional blast. Tandem and segmented duplication events were distinguished based on distance and chromosome distribution [88].

To estimate the duplication events of *TaZFP*s, we calculated Ka and Ks values of *TaZFP* duplicated gene pairs using the TBtools. Subsequently, the Ks values were used to approximate the duplication events according to T = Ks/2λ × 10^–6^ Mya, assuming a clock rate (λ) of 6.5 × 10^−^^9^ for synonymous substitutions [89].

### 4.4. Identification of Gene Structure, Conserved Motifs, and Cis-Acting Regulatory Elements

Intron and exon start and stop site information was obtained from the GFF3 annotation file of *T. aestivum*. The conserved motifs of the four TaZFP subfamilies were predicted using the Multiple Em for Motif Elucidation (MEME) website server (http://meme-suite.org/index.html, accessed on 17 May 2022), and the subfamily recognition motifs were retained. The specific parameters were set as follows: the number of sequence occurrences was any number of reputations (anr), the motif width was 6-200 amino acids, and other default settings were maintained. Motifs prediction results confirm the type of conserved structural domains via the SMART server (https://smart.embl.de/, accessed on 21 May 2022) [90]. To obtain the CAREs of *TaZFP*s, a 2-kb segment upstream of each gene’s transcription start site (TSS) was taken and used for prediction analysis using the PlantCARE database (http://bioinformatics.psb.ugent.be/webtools/plantcare/html/, accessed on 23 May 2022). Identified cis-acting elements are classified according to their function. Gene structure, conserved motifs, and cis-acting regulatory elements are analyzed and visualized by the tidyverse, ggplot2, and gggenes R packages.

### 4.5. GO Enrichment and Protein Interaction Network Establishment

GO annotations of *TaZFP*s were analyzed on agriGO (http://systemsbiology.cau.edu.cn/agriGOv2/index.php, accessed on 27 May 2022), and enrichment results were visualized using R (3.9.0). Protein-protein interactions (PPIs) were predicted using STRING database 11.5 (https://string-db.org/, accessed on 29 May 2022) and displayed on SCImago Graphica Beta 1.0.18, where interaction networks were screened with the criterion of combined scores > 0.7 [91]. The protein-protein interaction network of TaZFP was predicted with high confidence (0.700) and visualized using Cytoscape.

### 4.6. Gene Expression Analysis

To analyze the expression pattern of the *TaZFP*s under different treatments and different tissues, processed expression data were obtained from *T. aestivum* Omics 1.0 (http://wheatomics.sdau.edu.cn/, accessed on 10 July 2022) [92]. Specifically, for the expression profiles at different treatment times, two biological replicates were obtained for leaf tissue of *T. aestivum* seedlings after 1 h and 6 h of DS and HS treatments using transcriptomic data reported by Liu et al. [44]. For the expression profiles at the tissue level under DS and HS treatments, high-throughput RNA seq data (accession number: PRJNA358808) were used to analyze the differential expression patterns of leaf, root, and grain tissues of two cultivars (resistant and susceptible) under stress treatments (DS, HS, and DS+HS). The differential expression patterns of DS, HS, and DS+HSTPM (transcripts per million) values of the TaZFPs are shown in Table S8. Heatmaps were plotted by the R (3.9.0).

### 4.7. Plants Material and Culture

Based on GO analysis and abiotic stress expression heatmap results, drought-treated *T. aestivum* leaves were selected as the primary abiotic stress study. The bread *T. aestivum* cultivar Jimai 22 was used throughout the study. The seeds were placed in 10 cm × 10 cm perforated pots and incubated in an incubator (22 °C, 60% RH, 400 PPM CO_2_, 8 h dark, 16 h light) until the *T. aestivum* grew to the three-leaf stage. The cut-off water treatment was used as drought stress, and seedlings under normal growth conditions (22 °C, watered) were used as control. Leaves from the 3-day and 6-day drought treatments were collected, frozen immediately in liquid nitrogen, and stored at −80 °C for further studies [26,93]. All experiments were performed in parallel with three biological replicates at each specific time point.

### 4.8. RNA Extraction and qRT-PCR Analysis

Total RNA was extracted from each frozen sample using TRNzol Universal Reagent (Tiangen Biotech Co., Ltd., Beijing, China). cDNA synthesis was performed in one step according to the instructions of the TRUEscript 1st Strand cDNA Synthesis Kit (Aidlab Biotechnologies Co., Ltd., Beijing, China). Quantitative analysis was performed on a QuantStudio^TM^ 1 Real-Time PCR Instrument (Applied Biosystems, Foster City, CA, United States) using TaKaRa TB Green^TM^ Premix Ex TaqTM II (Takara Biotechnology Co., Ltd., Beijing, China) with a reference fluorescence of SYBR. According to a previous study, the actin gene was used as an internal control. Each reaction system was carried out in 25 µL of the mixture. The reaction mixture contained the following reagents: 1.0 µL cDNA, 2 µL of each primer pair, 12.5 µL SYBR, and 9.5 µL ddH_2_O. Gene-specific primers are shown in Table S9. qRT-PCR cycling parameters were 40 cycles at 95 °C for 15 s and 60 °C for 1 min, with a ramp-up and ramp-down rate of 1.6 °C/s. At the end of the reaction, samples were slowly heated from 60 °C to 95 °C at 0.15 °C/s, and melting curves were generated by continuous fluorescence monitoring. All reactions were repeated three times to ensure reproducible results. The qRT-PCR results were analyzed using the 2^−∆∆CT^ method to estimate ploidy changes in the expression levels of the genes of interest using 3-day and 6-day control treatments [94].

## 5. Conclusions

ZFPs, as one of the critical transcription factors, are involved in various physiological processes, such as seed development, plant growth, and biotic and abiotic stress responses. In this study, we investigated the physicochemical properties, phylogeny, chromosome distribution, gene duplication, covariance, gene structure, conserved motif, stress-related CAREs, GO enrichment, protein-protein interactions, expression patterns of 269 *TaZFP*s (9 TaC2H2, 38 TaC3HC4, 79 TaCCCH, and 143 TaPHD) in *T. aestivum*. TaC2H2, TaC3HC4, TaCCCH, and TaPHD genes were classified into 4, 7, 12, and 14 classes based on the phylogenetic tree constructed from *ZFP* genes of *T. aestivum*, *A*. *thaliana*, *T.* *dicoccoides,* and *O. sativa*, respectively, according to the ZFP subfamily classification. Meanwhile, we analyzed the number distribution and density heterogeneity of the four TaZFP subfamilies at subgenomic and chromosome levels. We identified 143 fragment duplication events and 7 tandem duplication events in *TaZFP*s, indicating that fragment duplication played a dominant role in the evolution of *TaZFP*s. Furthermore, the synteny analysis and Ka/Ks results between *T. aestivum* and the other three representative plants further indicated that four *TaZFP* gene subfamilies experienced strong purifying selection. Subsequently, four gene subfamilies were identified with specific motifs and diverse gene structures. Many abiotic stress-related CAREs were identified in the promoters of *TaZFP*s. GO enrichment results showed that all *TaZFP*s were annotated under the nucleic acid binding and metal ion binding. The expression patterns indicated that some *TaZFP*s were involved in response to DS and HS. Overall, this study provides comprehensive information on the sizeable *TaZFP* gene subfamilies, rarely studied on a subfamily scale, and will help identify particular *TaZFP* gene functions in further studies. Further, the characterization of this *T. aestivum* gene family, which is highly responsive to drought and heat, will undoubtedly provide new theoretical support and inspiration at the molecular level for use in agriculture, ecosystem studies, modelling, and other fields.

## Data Availability

All data are available on reasonable request to the corresponding author. Most of the data is sourced from publicly available databases.

## Supplementary Materials

The following supporting information can be downloaded at: https://www.mdpi.com/article/10.3390/plants11192511/s1, Figure S1: Phylogenetic tree, gene structure, and conserved motifs of TaZFPs. Figure S2: Conserved motifs of TaZFPs. Figure S3: *Cis*-acting regulatory elements of *TaZFP*s. Table S1: Details of the identified *TaZFP* genes. Table S2: Members of four TaZFP subfamilies phylogenetic tree. Table S3: Putative functions of the TaZFP in *T. aestivum*. Table S4: Ka/Ks ratios and divergence times of gene pairs in *T. aestivum*. Table S5: Orthologous pairs between *T. aesticum* and other plants. Table S6: *Cis*-acting regulatory elements (CAREs) in the promoter of *TaZFP* genes. Table S7: Gene ontology of the *TaZFP* genes in *T. aestivum*. Table S8: The expression levels of *TaZFP* genes of *T. aestivum* under drought stress. Table S9: Primer sequences for qRT-PCR.
