# Supplementary figures and images for: Genome-Wide Identification and Expression Analysis of the Zinc Finger Protein Gene Subfamilies under Drought Stress in Triticum aestivum
Source: Plants (Basel). 2022 Sep 26;11(19):2511. doi: 10.3390/plants11192511 (PMC9572532; doi:10.3390/plants11192511)

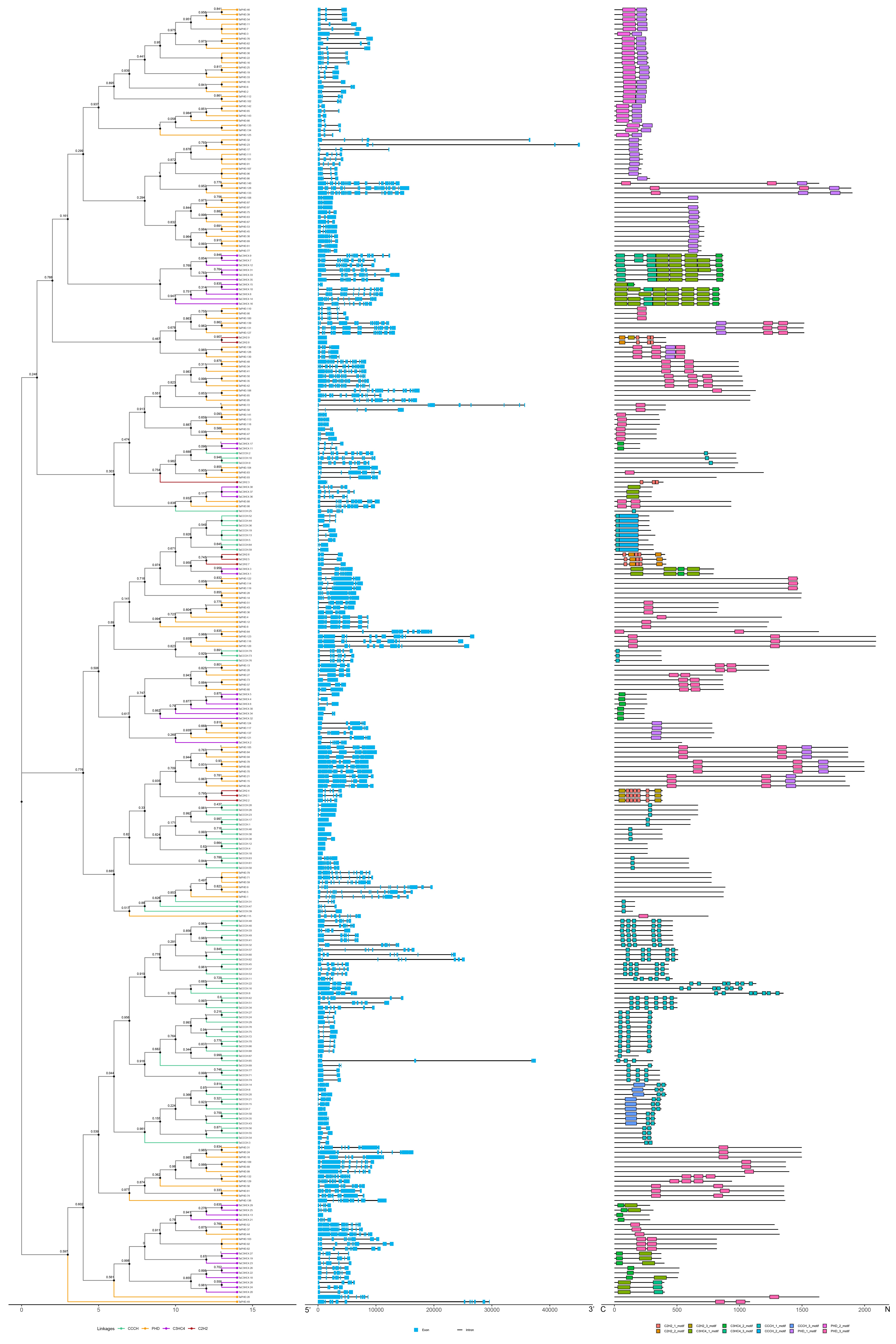

Supplement: Supplementary file 1 [file plants-11-02511-s001.zip › Figure S1.jpg]

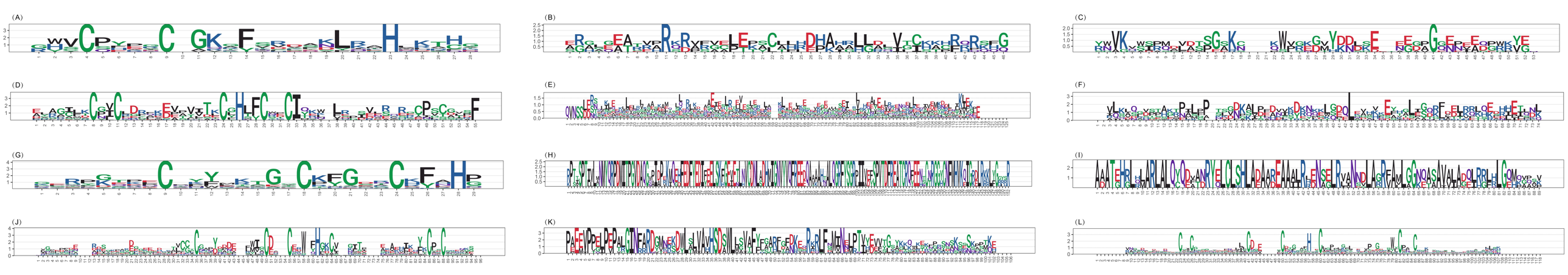

Supplement: Supplementary file 1 [file plants-11-02511-s001.zip › Figure S2.jpg]

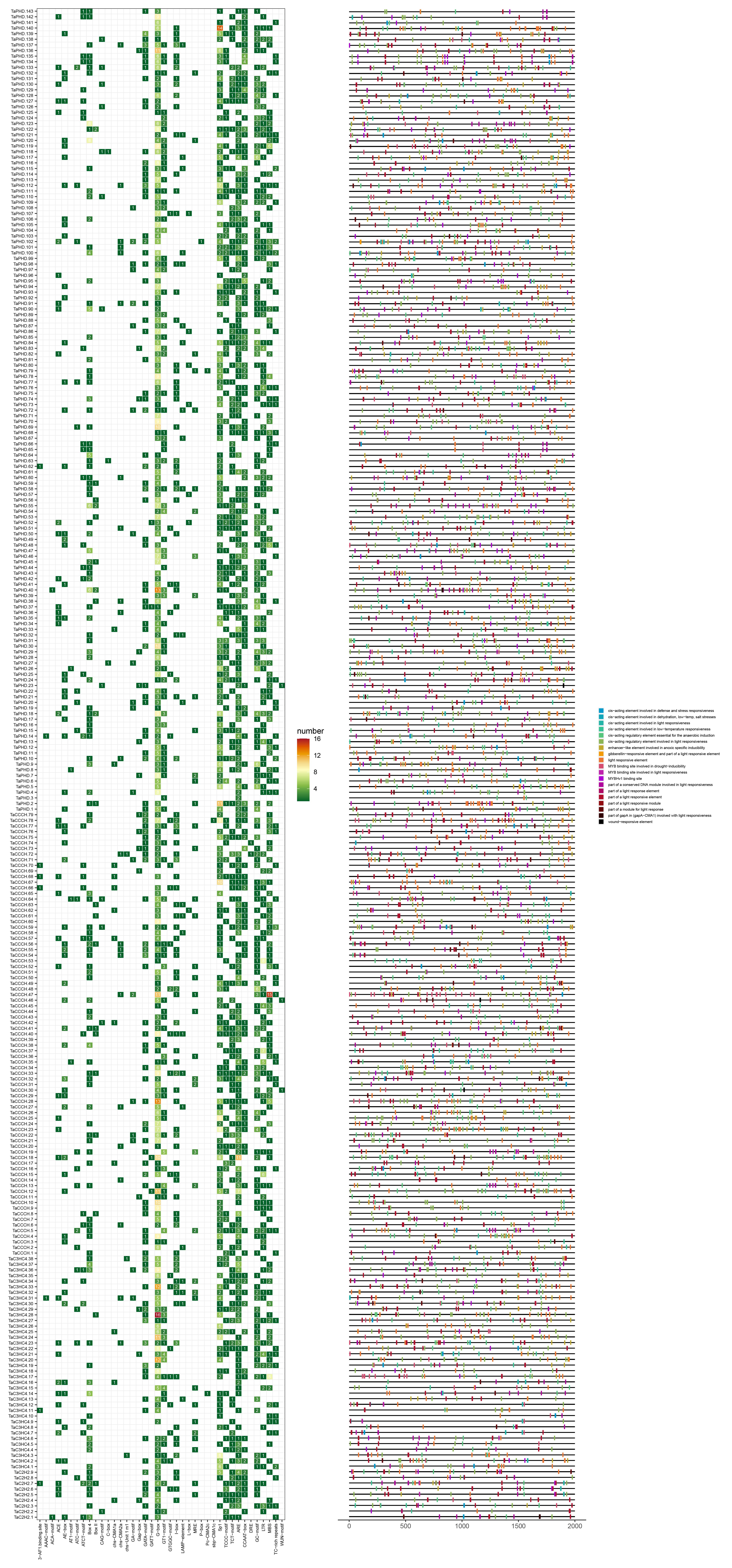

Supplement: Supplementary file 1 [file plants-11-02511-s001.zip › Figure S3.jpg]
